# Letter to the Editor regarding: “Pre- and post-analytical guidelines for the microscopic diagnosis of melanoma: recommendations from the Brazilian Society of Pathology” – Reply^؆^

**DOI:** 10.1016/j.abd.2025.501272

**Published:** 2026-01-19

**Authors:** José Cândido Caldeira Xavier-Júnior, Karina Munhoz de Paula Alves Coelho, Mariana Petaccia de Macedo, Rute Facchini Lellis, Nathanael de Freitas Pinheiro Junior, Robledo Fonseca Rocha, Comitê de Dermatopatologia da Sociedade Brasileira de Patologia

**Affiliations:** a Instituto de Patologia de Araçatuba, Araçatuba, SP, Brazil; b Faculty of Medicine, Centro Universitário Católico Unisalesiano, Araçatuba, SP, Brazil; c Postgraduate Program in Pathology, Faculty of Medicine, Universidade Estadual Paulista, Botucatu, SP, Brazil; d Centro de Diagnósticos Anátomo-Patológicos (CEDAP), Joinville, SC, Brazil; e Instituto Nacional de Ciência e Tecnologia em Biologia do Câncer Infantil e Oncologia Pediátrica ‒ INCT BioOncoPed, Porto Alegre, RS, Brazil; f Department of Pathology, Rede D’Or/São Luiz Hospital, São Paulo, SP, Brazil; g Laboratory of Pathology, Hospital da Santa Casa de São Paulo, Santa Casa de Misericórdia de São Paulo, São Paulo, SP, Brazil; h Imagepat Anatomia Patológica Ltda, Salvador, BA, Brazil; i Centro de Ciências da Saúde, Universidade Federal de Roraima, Boa Vista, RR, Brazil; j Laboratório de Patologia de Roraima, Boa Vista, RR, Brazil

Dear Editor,

We read with great interest the letter from Dr. Cunha regarding our recently published guideline.^1^ Both the College of American Pathologists^2^ and the NCCN^3^ strongly discourage the use of frozen sections for most melanocytic lesions when standard clinical margins can be achieved without significant limitations. They emphasize that accurate melanoma diagnosis continues to rely on Hematoxylin and Eosin (H&E) sections from formalin-fixed, paraffin-embedded tissue, supplemented, when appropriate, by immunohistochemistry,^2^, ^3^, ^4^ since melanoma represents a heterogeneous group of tumors with multiple histologic subtypes and distinct biological behaviors.^4^

The recommended peripheral surgical margins and methods for their assessment in head and neck melanoma and lentigo maligna remain subjects of considerable debate. This uncertainty largely reflects the lack of consistent, well-designed prospective studies addressing the management of these challenging lesions. A recent critical review identified a very high risk of bias (97.9%) among studies evaluating Mohs Micrographic Surgery (MMS) for melanoma.^5^

Although robust validation for the use of MMS in melanocytic lesions is still lacking, recent advances in this field deserve recognition. According to the latest NCCN guidelines for cutaneous melanoma, MMS ‒ or other techniques that enable comprehensive margin assessment ‒ may provide equivalent or even superior local control for certain pT1a melanomas in selected sites, particularly on the face, ears, and acral areas.^3^ It is essential, however, that potential candidates be carefully selected and thoroughly counseled. The successful use of these techniques, which apply only to a small subset of melanoma patients, requires a well-trained and experienced multidisciplinary team. It is important to highlight that, considering melanomas arising in skin with extensive cumulative sun damage, histopathologic interpretation can be especially challenging. This difficulty arises from actinic melanocytic alterations and ill-defined tumor margins with “skip” areas^4^ ‒ factors that may be further compounded by technically suboptimal slides.

Finally, as also acknowledged by the NCCN,^3^ no prospective comparative studies have yet evaluated the different excision methods for melanoma. This gap leaves the topic open for continued scientific discussion. Future prospective, independent, and well-designed studies are needed to clarify the potential role of MMS and other surgical techniques in the management of melanocytic lesions.

## Financial support

None declared.

## Authors’ contributions

José Cândido Caldeira Xavier Junior: Contributed to the design and planning of the study; drafting, editing, or critically reviewing the manuscript for important intellectual content; active participation in research supervision; critical review of the literature; and approval of the final version of the manuscript.

Karina Munhoz de Paula Alves Coelho: Contributed to the design and planning of the study; drafting, editing, or critically reviewing the manuscript for important intellectual content; active participation in research supervision; critical review of the literature; and approval of the final version of the manuscript.

Mariana Petaccia de Macedo: Contributed to the design and planning of the study; drafting, editing, or critically reviewing the manuscript for important intellectual content; active participation in research supervision; critical review of the literature; and approval of the final version of the manuscript.

Rute Facchini Lellis: Contributed to the design and planning of the study; drafting, editing, or critically reviewing the manuscript for important intellectual content; active participation in research supervision; critical review of the literature; and approval of the final version of the manuscript.

Nathanael de Freitas Pinheiro Junior: Contributed to the design and planning of the study; drafting, editing, or critically reviewing the manuscript for important intellectual content; active participation in research supervision; critical review of the literature; and approval of the final version of the manuscript.

Robledo Fonseca Rocha: Contributed to the design and planning of the study; drafting, editing, or critically reviewing the manuscript for important intellectual content; active participation in research supervision; critical review of the literature; and approval of the final version of the manuscript.

## ORCID ID

Mariana Petaccia de Macedo: 0000-0002-0434-7605

Rute Facchini Lellis: 0000-0001-7690-0513

Nathanael de Freitas Pinheiro Junior: 0000-0003-0691-3006

Robledo Fonseca Rocha: 0009-0000-0083-8434

## Research data availability

Does not apply.

## Conflicts of interest

None declared.
